# Box-Counting Dimension Revisited: Presenting an Efficient Method of Minimizing Quantization Error and an Assessment of the Self-Similarity of Structural Root Systems

**DOI:** 10.3389/fpls.2016.00149

**Published:** 2016-02-18

**Authors:** Martin Bouda, Joshua S. Caplan, James E. Saiers

**Affiliations:** ^1^Saiers Lab, School of Forestry and Environmental Studies, Yale UniversityNew Haven, CT, USA; ^2^Department of Ecology, Evolution and Natural Resources, Rutgers, The State University of New JerseyNew Brunswick, NJ, USA

**Keywords:** root architecture, fractal dimension, numerical optimization, self-similarity, plant root growth, code:MATLAB

## Abstract

Fractal dimension (FD), estimated by box-counting, is a metric used to characterize plant anatomical complexity or space-filling characteristic for a variety of purposes. The vast majority of published studies fail to evaluate the assumption of statistical self-similarity, which underpins the validity of the procedure. The box-counting procedure is also subject to error arising from arbitrary grid placement, known as quantization error (QE), which is strictly positive and varies as a function of scale, making it problematic for the procedure's slope estimation step. Previous studies either ignore QE or employ inefficient brute-force grid translations to reduce it. The goals of this study were to characterize the effect of QE due to translation and rotation on FD estimates, to provide an efficient method of reducing QE, and to evaluate the assumption of statistical self-similarity of coarse root datasets typical of those used in recent trait studies. Coarse root systems of 36 shrubs were digitized in 3D and subjected to box-counts. A pattern search algorithm was used to minimize QE by optimizing grid placement and its efficiency was compared to the brute force method. The degree of statistical self-similarity was evaluated using linear regression residuals and local slope estimates. QE, due to both grid position and orientation, was a significant source of error in FD estimates, but pattern search provided an efficient means of minimizing it. Pattern search had higher initial computational cost but converged on lower error values more efficiently than the commonly employed brute force method. Our representations of coarse root system digitizations did not exhibit details over a sufficient range of scales to be considered statistically self-similar and informatively approximated as fractals, suggesting a lack of sufficient ramification of the coarse root systems for reiteration to be thought of as a dominant force in their development. FD estimates did not characterize the scaling of our digitizations well: the scaling exponent was a function of scale. Our findings serve as a caution against applying FD under the assumption of statistical self-similarity without rigorously evaluating it first.

## 1. Introduction

**Figure 1:**
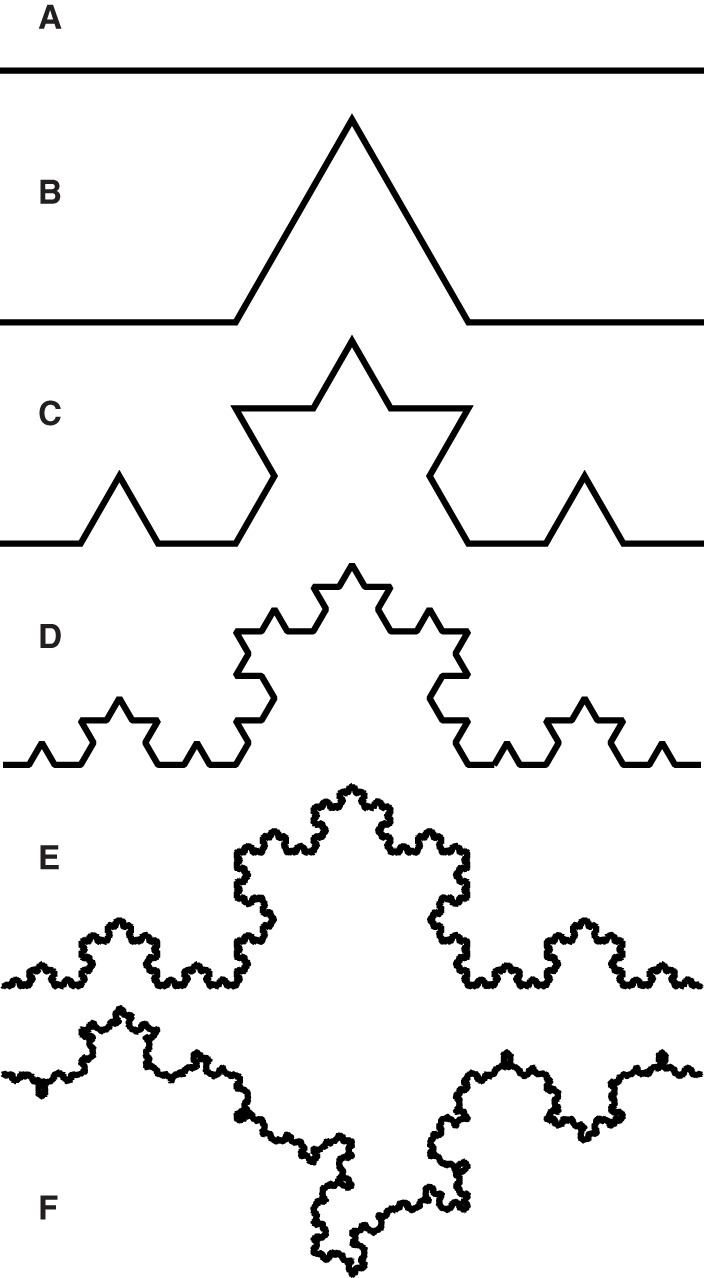
**Construction of the Koch curve, following Falconer (2003)**. Each interval **(A)** is divided evenly into three and the middle section is replaced by the complementary two sides of an equilateral triangle **(B)**. The process is repeated for each newly created interval, yielding the second **(C)**, third **(D)**, and *n*^th^ iterations. The Koch curve is the limit approached as *n* → ∞. The limit curve can be subdivided into four quarters, each an exact copy of the whole, scaled down by a factor of three. The curve is thus self-similar with a similarity dimension of log(4)/log(3). Even with *n* = 10 **(E)**, zooming in on the pinnacle of the curve by a factor of three yields an image visually indistinguishable from the largest magnification five times over, meaning the curve is approximately self-similar over a finite range of scales. Following the same construction, but randomly choosing the side of the old interval on which each new pair of intervals is placed, yields one of many “statistically self-similar” curves **(F)**. These cannot be divided into sets of identical copies; rather, their parts are scaled random variations on the whole and they only conform to a fractal dimension on average.

Fractal dimensions (FD) are metrics useful in characterizing the geometry of sets too irregular to be described in more classical ways that nevertheless exhibit sufficient fractal regularity (Falconer, 2003). A FD will hold useful information on the geometry of such sets, while Euclidean measures may not. For example, a Koch curve (see Figures 1A–E for construction) has infinite length, zero area, and a finite similarity (fractal) dimension of log(4)∕log(3). Although a Koch curve is too irregular to be well described by Euclidean measures, its self-similarity allows for a simple description of its scaling properties via this FD. An even less regular example is a random Koch curve (Figure 1F), which does not consist of scaled images of itself, but rather of stochastic variations on these. The placement of its details at any scale is random, and can be highly variable from one implementation to the next, but the rate at which detail emerges as its image is magnified is the same as for the original Koch curve. It can thus still be described by the same FD on average and we say that it exhibits “statistical self-similarity.” This example illustrates that “fractal” and “self-similar” are not synonyms: self-similarity, or statistical self-similarity, is just one mode of fractal regularity.

Following their popularization (e.g., Mandelbrot, 1967, 1983), the concepts of fractal geometry have been applied to diverse natural phenomena. Objects considered “natural fractals” also tend to defy description using classical geometry, to exhibit meaningful detail over a large range of scales, and to lend themselves to informative description using some FD, at least in a statistical sense, over some finite range of scales (Mandelbrot, 1967; Falconer, 2003; Halley et al., 2004). In short, objects are worth studying *as* fractals if they show detail at too many scales to be well approximated by classical geometric sets but that detail is sufficiently consistent to be approximated with fractal models.

### 1.1. FD estimation for root systems

FD estimates have been obtained for various plant structures in order to quantify their space-filling characteristic, complexity, and branching intensity (Tatsumi et al., 1989; Lynch and Vanbeem, 1993; Berntson, 1994; Berntson and Stoll, 1997; Eshel, 1998; Dzierzon et al., 2003; Da Silva et al., 2006). Estimated FD has been related to canopy light interception (Dutilleul et al., 2008, 2015) and root system soil exploration efficiency (Walk et al., 2004). It has been used with a view to draw inferences about resource acquisition of individuals (Eghball et al., 1993; Nielsen et al., 1997, 1999; Masi and Maranville, 1998; Manschadi et al., 2008) and plant communities (Dannowski and Block, 2005). Others have used it to characterize plant-soil interactions, quantifying plant response to drought (Wang et al., 2009), soil saturation (Pierce et al., 2013), mycorrhizal colonization (Yang et al., 2014), or salinity (Subramanian et al., 2015). Still others have used it with stated goals like linking branching intensity to water use efficiency (Bari et al., 2004), elucidating interspecific differences in life-history and growth strategies (Oppelt et al., 2000), phenotyping (Grift et al., 2011), assessing canopy complexity across successional stages (Aagaard and Hartvigsen, 2014), measuring the effect of plant complexity on invertebrate habitat heterogeneity (Dibble and Thomaz, 2009), assessing competition in an intercropping system (Izumi and Iijima, 2002), or evaluating the effect of different invasive plant removal techniques on subsequent colonization (Barto and Cipollini, 2009; Ferreiro et al., 2013).

On reviewing plant FD estimation studies (Supplementary Table 1) we found that several of the known methodological issues with the box-counting procedure (Reeve, 1992; Berntson, 1994; Berntson and Stoll, 1997; Foroutan-pour et al., 1999; Gonzato et al., 2000; Halley et al., 2004; Da Silva et al., 2006) are often either ignored or addressed in an unsystematic way in the plant science literature. These issues can be grouped around two general headings: the statistical self-similarity of plant parts (in this study, coarse root systems) and the reduction of quantization error. In the following two sections, we introduce each problem cluster in turn, as context for the goals of this work, which are stated in the last part of the introduction.

### 1.2. Box-count dimension and statistical self-similarity of root systems

**Figure 2:**
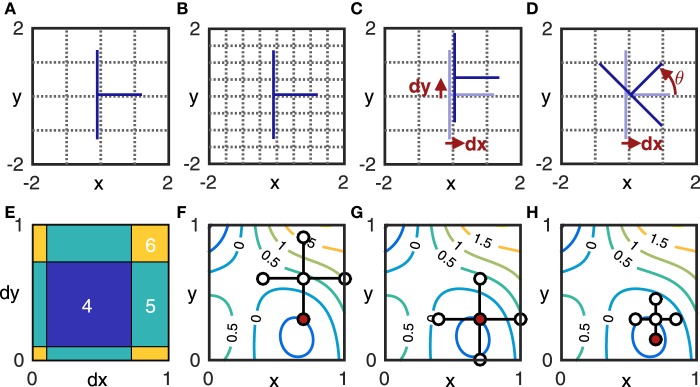
**Components of numerical methods used in this study**. **(A,B)** show box-counting in 2D on an idealized skeletonized root in blue: at box size *s* = 1 the root intersects *N* (*s*) = 6 boxes **(A)**, at *s* = 1∕2, *N* (*s*) = 9 **(B)**. **(C,D)** show the effect of placement on the box-count with *s* = 1: translation by *dx* and *dy* yields *N* = 4; translation by *dx* and rotation by θ yields *N* = 3. Taking *N* = 3 as the minimum value, the box-count estimates *N*_ϵ_ in **(A,C)** include quantization error ϵ_*A*_ = 3 and ϵ_*C*_ = 1, respectively. **(E)** shows the box-count *N*_ϵ_ as a function *J* (*dx, dy*) at θ = 0. It generalizes **(C)** to show the box-count values for all combinations of 0 < *dx* < 1 and 0 < *dy* < 1. Note that the function jumps by integer values and is thus not continuously differentiable, but exhibits the following symmetry: *J* (*dx* + *a, dy*, θ) = *J* (*dx, dy* + *a*, θ) = *J* (*dx, dy*, θ) for any integer *a*, since a translation of the grid in any direction by the grid-size *s* yields the same grid. The same symmetry is obtained for rotation by multiples of π ∕ 2. **(F–H)** illustrate pattern search on the contour plot of an unknown function *Z* = *f* (*x, y*). Given a starting point (*x*_0_, *y*_0_) to serve as the center for a five-point stencil and a step-size Δ*v*, the function is evaluated in step one **(F)** at the center and four outlying points (*x*_0_ ± Δ*v, y*_0_ ± Δ*v*). The lowest value of *Z* (indicated in red) is found at (*x*_0_, *y*_0_ − Δ*v*), which thus becomes the stencil center for the next step. In step two **(G)**, the function is again evaluated at each of the five points of the new stencil. The minimum value (red) is found at the center this time, so the stencil is not moved for the next step, but the step-size is reduced to Δ*v*∕2. In step three **(H)** the function is evaluated at the five points of the new stencil and a new minimum is identified. This process continues until the step-size reaches a chosen lower bound.

The FD used in all the studies we reviewed and most commonly applied to natural fractals is box-counting dimension; it quantifies the rate at which an object's geometrical details develop at increasingly fine scales (Falconer, 2003). In each step of box-counting, an object is covered by a grid of boxes of side length *s* and the number of boxes *N* intercepted by the object is found (see Figures 2A,B). The box-count dimension *D* is defined as

(1) $$\begin{array}{l} D=\underset{s\to 0}{\lim}\frac{-\log\left(N\left(s\right)\right)}{\log\left(s\right)}\text{.} \end{array}$$

For mathematical sets for which this limit does not exist, the box-counting dimension is not well defined and only the lower and upper box-count dimensions may be estimated (as the lower and upper limits, respectively; Falconer, 2003).

Natural objects exhibit detail over a finite range of scales *s* ∈ (*s*_*L*_, *s*_*U*_), with lower limit *s*_*L*_ and upper limit *s*_*U*_. Thus, for natural objects, as *s* → 0 in the region *s* < *s*_*L*_, the ratio log(*N*) ∕ log(*s*) tends to converge on their (more commonly understood) topological dimension *D*_*T*_. That is, for a skeletonized root system represented as a finite set of line segments, *D* → 1 as *s* → 0, since *D*_*T*_ = 1 for line segments. This is not an aberration: one is the correct box-count dimension of a root system represented as a finite set of line segments. At the other end of the range, since a single box covers the entire object in the region *s* > *s*_*U*_, the ratio takes values of 1∕*s* and it simply converges to 0 as *s* → ∞. To apply the box-counting procedure to such an object as a means of estimating its fractal dimension, one must therefore locate the range *s* ∈ (*s*_*L*_, *s*_*U*_), over which the object actually exhibits more detail as *s* diminishes, and look at the slope of the log(*N*) vs. log(1 ∕ *s*) relationship there. The idea is to use the class of statistically self-similar fractals as a model for the object over this range of scales, which implies a constant mean rate of emergence of detail across scales and thus allows, by assumption, using the mean slope of the relationship over this region as a stand-in for the limit value in Equation (1), effectively extrapolating it down to 0. The procedure measures the box-count dimension not for the finite set itself, but its fractal approximation.

Let us call those natural objects for which some range of scales *s* ∈ (*s*_1_, *s*_2_), where *s*_*L*_ ≤ *s*_1_ < *s*_2_ ≤ *s*_*U*_, can be found, such that log (*N*(*s*)) ∝ log (1 ∕ *s*) ∀*s* ∈ (*s*_1_, *s*_2_) to first approximation, “statistically self-similar” natural fractals. Such terms are sometimes used for natural objects (e.g., Mandelbrot, 1967; Berntson and Stoll, 1997), though not necessarily with a single widely accepted definition (Falconer, 2003). Our definition here is consistent with previous usage and identifies natural objects that can be well approximated as self-similar mathematical fractals over a finite range of scales. For such objects, a fractal box-counting dimension can be estimated from the slope of the log(*N*) vs. log(1 ∕ *s*) relationship, which is most commonly done by least-squares regression. Objects that lack this property will not yield a single, constant slope when subjected to box-counting. The less well this fractal model approximates the object's properties, i.e., the more the slope of the relationship changes lawfully over the investigated range of scales, the less information is contained in any single average slope ultimately found. In other words, objects that do not fulfill this definition are not suitable for idealization as statistically self-similar mathematical fractals and box-count dimensions obtained for them will not informatively describe their scaling characteristics.

Studies that actually employ a rigorous test of statistical self-similarity on root system datasets are scarce and may find it absent (Berntson and Stoll, 1997). Most studies use least-squares regression to estimate box-count dimension and a number of authors use the associated *R*^2^ (coefficient of determination) to evaluate the strength of the relationship or to support claims about the self-similarity, statistical self-similarity, or “fractal nature” of the plant parts investigated (Oppelt et al., 2000; Lontoc-Roy et al., 2005; Han et al., 2008; Yang et al., 2014). As Reeve (1992) pointed out, however, the independence assumption underlying regression is violated in the box-counting procedure and thus, when the slope of log(*N*) vs. log(1 ∕ *s*) is estimated using regression, the error estimate is deflated, giving an inflated *R*^2^ and a false sense of precision. The use of *R*^2^ on box-count regressions is an insufficient means of evaluating the statistical self-similarity of a root system.

Many other studies do not attempt to evaluate the fractal model for their root dataset at all. More recent studies sometimes justify their use of box-count dimension, their assumption of statistical self-similarity, or ascribing “fractal geometry” to root systems by reference to the earlier ones (Barto and Cipollini, 2009; Grift et al., 2011; Pierce et al., 2013), which seems to incorrectly suggest that statistical self-similarity has been shown to hold for root system data generally. Moreover, earlier studies often use *R*^2^ to validate their fractal model, or else base their claims on purely theoretical grounds. Some suggest, for example, that root systems' complexity alone makes them suitable to fractal description (Nielsen et al., 1997). Again, the complexity of a root system by itself is an incomplete justification for using a fractal model, since even a complex object may be better described by classical than by fractal geometrical models, depending on how its complexity is organized.

Others rely on the idea that sufficient repetitive branching will give the resulting systems a level of self-similarity (Eshel, 1998; Dannowski and Block, 2005), which is justified in principle. Repetition of the same steps in the generative process is important to the emergence of strict or statistical self-similarity in both mathematical (as illustrated in Figure 1) and natural (Brodkey, 1966) fractals and reiterative growth is commonly cited as a source of complexity in plant architecture (Barthélémy and Caraglio, 2007; Costes et al., 2013). If we can find self-similarity in some part of plant anatomy, this may suggest that its developmental process is dominated by reiteration over a great range of scales, an idea exploited by so-called fractal root system models (van Noordwijk et al., 1994; Ozier-Lafontaine et al., 1999). On the other hand, the relative importance of reiteration in the root system developmental process, or its level of ramification, may not be sufficient to substantiate a naïve expectation of statistically self-similar scaling.

Moreover, the relation between stochastic reiterative growth and statistical self-similarity is not entirely straightforward. If plant roots develop mainly by stochastic reiterative growth over a sufficient range of scales, yielding structures akin to statistically self-similar mathematical fractals, then the resulting log(*N*) vs. log(1 ∕ *s*) plots obtained from box-counting should strictly follow a linear model. That is to say, the box-count data will be generated as log(*N*) = *D* log(1 ∕ *s*) + ϵ, where the error ϵ ~ *N*(0, σ). Construction of a set in this manner is a sufficient, but not necessary, condition for observing this scaling pattern, implying that we can refute stochastic reiterative growth should the model be a poor fit, but we cannot confirm it just by fitting the model to the data successfully.

Even if it can be shown that an object was not generated by reiteration (e.g., through an analysis of residuals as in Berntson and Stoll, 1997, and this study), it is still possible that its log(*N*) vs. log(1 ∕ *s*) plot will be linear to a first approximation over some range, a condition described as “apparent fractality” (Hamburger et al., 1996; Halley et al., 2004). Pragmatically speaking, a set exhibiting apparent fractality is sufficiently statistically self-similar that treating it as a fractal may be informative even if the mechanisms that generated it are not “fractal” in any meaningful sense. Indeed, a root system need not be developed strictly by stochastic reiteration, like the random Koch curve, for it to scale roughly as though it was. Since a single value may be representative of the slope of a non-linear curve over some range, we may still estimate its box-count dimension in such a case, as shown by Mandelbrot (1967) for geographical frontiers. But the further a root system deviates from statistical self-similarity, the less representative any one box-count dimension value will be of its complexity pattern until, at some point, using Euclidean measures (such as total root length, length density, or branching frequency) to characterize its form and complexity will become preferable. Evaluating the hypothesis of statistical self-similarity on root systems is thus a worthwhile step in any study wishing to use box-count FD estimates to describe root systems.

### 1.3. Quantization error

Another issue that is known (Foroutan-pour et al., 1999; Gonzato et al., 2000; Da Silva et al., 2006) but not comprehensively addressed in the literature on natural fractals, is that of quantization error (QE). QE comes from miscounting the boxes of a certain size necessary to cover an object. This possibility arises because measuring an object at a given scale in FD estimation entails finding the *minimum* number of boxes of a given size needed to cover it. By contrast, the box-counting procedure finds the number of boxes intercepted by an object using an *arbitrary* grid, yielding a value *N*_ϵ_ = *N* + ϵ, where *N* is the true count and ϵ is quantization error. The error (illustrated in Figures 2C,D) is due to the position and orientation (collectively the placement) of the arbitrary grid; its distribution is discrete and supported on a finite interval of non-negative integers. As such, quantization error violates the assumptions of linear regression, although it also affects local slope estimates (Da Silva et al., 2006). Estimation of fractal dimension from pixelated images leads to special cases of quantization error (Gonzato et al., 2000; Halley et al., 2004; Lontoc-Roy et al., 2005), depending on the nature of the digitization process. Failing to account for quantization error can have a significant effect on the accuracy of the FD estimation as a whole (Da Silva et al., 2006, as well as our results). Nevertheless, quantization error has often been ignored in the estimation of root system FD (Supplementary Table 1).

More recent studies have attempted to reduce QE (Foroutan-pour et al., 1999; Lontoc-Roy et al., 2005, 2006; Da Silva et al., 2006; Han et al., 2008; Dutilleul et al., 2015; Subramanian et al., 2015) or at least used software capable of addressing it (Pierce et al., 2013). Such studies translate the grid either systematically (Foroutan-pour et al., 1999; Szustalewicz, 2007) or randomly (Gonzato et al., 2000; Karperien, 2007–2012) to sample the grid domain a certain number of times at each box size and choose the minimum box-count achieved. The approach is sure to reduce QE in principle, but the large computational expense involved means that relatively few possible grids are usually attempted in practice (Lontoc-Roy et al., 2005, 2006; Han et al., 2008; Dutilleul et al., 2015; Subramanian et al., 2015). This state-of-the-art “brute force” approach might thus be ineffective with too few grids and too computationally inefficient to be implemented on a sufficient scale to deal with the error reliably. Also, grid translation alone may be an insufficient means of reducing QE, as the literature contains evidence both for (Gonzato et al., 2000) and against (Da Silva et al., 2006) the independent importance of grid rotation.

### 1.4. Study aims

Within this framework, this work will use a dataset of 36 digitized coarse root systems, aiming to

1. establish the significance of QE due to grid translation and rotation in root system FD estimation,
2. demonstrate an efficient method of reducing QE, and
3. use the low-error box-count estimates obtained to evaluate the hypothesis that the digitizations in our dataset are sufficiently statistically self-similar for a box-counting FD estimate to provide a first approximation of their scaling properties.

While our focus is on root systems, the methods we present can equally well be applied to box-counting on other objects.

## 2. Materials and methods

### 2.1. Data collection and format

Root architectural datasets came from plants that were excavated and digitized in the field. Individuals from six shrub species that are common in eastern North American deciduous forests were identified in natural areas of central New Jersey, USA. The species were *Berberis thunbergii* D.C. (Japanese barberry), *Lonicera maackii* (Rupr.) Maxim. (Amur honeysuckle), *Rubus phoenicolasius* Maxim. (wineberry), *Viburnum dentatum* L. (arrowwood viburnum), *Lindera benzoin* L. (spicebush), and *Rubus allegheniensis* Porter (common blackberry). These species were selected primarily because they represented a range of adaptation to soil nutrient conditions. Six individuals of each species were selected, yielding a total of 36 plants; their heights ranged from 0.6 to 2.8 m. After soil was removed with an AirKnife, root systems were secured in place and digitized in 3D using a Fastrack magnetic positioning system (Polhemus, Colchester, VT, USA) and PiafDigit software (UMR-PIAF, INRA, Clermont, France). Digitization methods followed Danjon et al. (1999). Briefly, we recorded the spatial coordinates of points at the ends of approximately linear segments; this was done for all roots whose basal diameters were greater than 2 mm. Diameters were measured manually at each recorded set of spatial coordinates, with non-circular cross sections approximated by the geometric mean of the widest axis length and that perpendicular to it. Topological information was recorded simultaneously with geometric data (Godin et al., 1999); the resulting dataset was a multi-scale tree graph (MTG), which are frequently used for describing root architecture in 3D (Dupuy et al., 2007; Danjon et al., 2013; Valdés-Rodríguez et al., 2013).

### 2.2. Box-count setup

**Figure 3:**
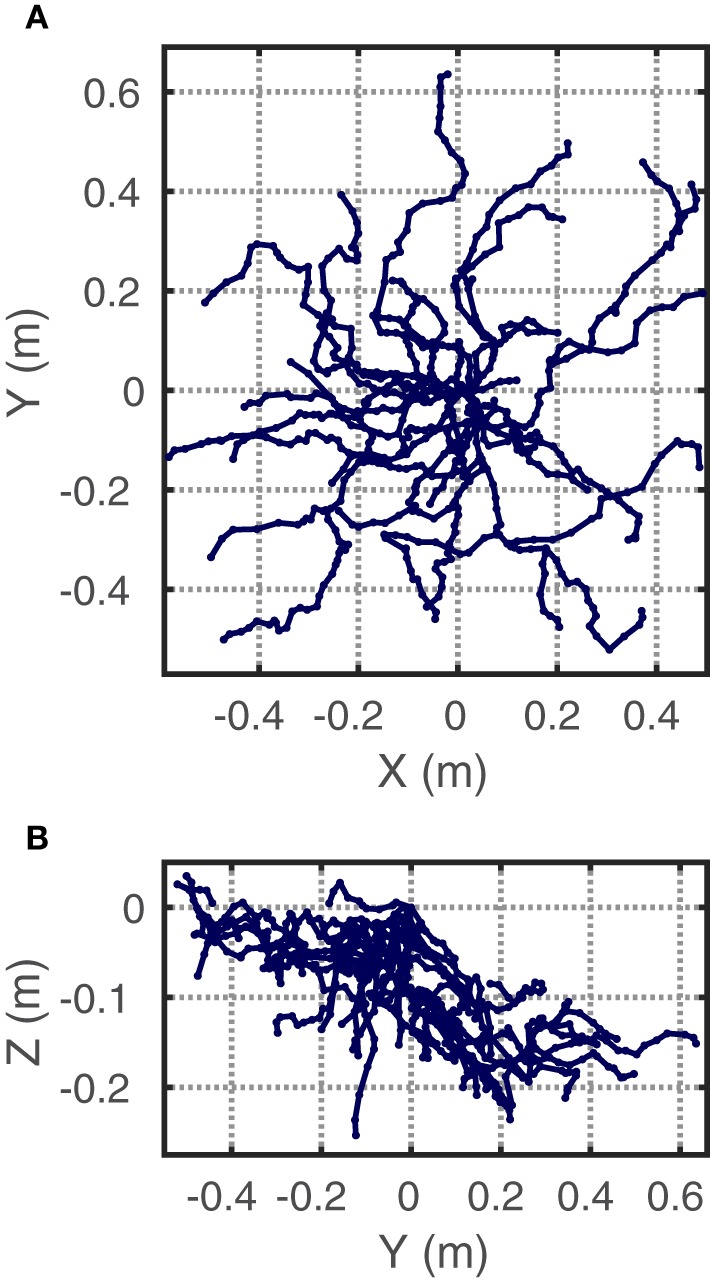
**Example of the coarse root system representations used for box-counting**. The data are piecewise linear interpolants constructed from the positional and topological data of a MTG of a *Berberis thunbergii* plant in **(A)** top- and **(B)** side-view. Grid origin set at root collar.

A piecewise linear interpolant of the connected data points in the MTG was used to represent each skeletonized root system (Figure 3). These sets were subjected to box-counting as follows. Maximum, minimum and intermediate 3D box sizes (*s* = 1, 2, 4, 8, and 16 cm) were chosen as successive powers of 2 covering the region where the plot of log(*N*) ∕ log(*s*) would usually be considered linear (Foroutan-pour et al., 1999; Halley et al., 2004). The box-counting algorithm found grid boxes intercepted by each line segment and recorded the number of boxes intercepted by the whole plant.

### 2.3. Grid transformations

Reducing quantization error despite using arbitrary grids entails imposing multiple grids by translation or rotation and choosing the lowest resulting box-count (Figures 2C,D). Our study uses a systematic brute-force sampling of the grid domain (Section 2.3.2) as a computational cost baseline. We contrast this common approach with an alternative one, in which the search for the grid that yields the lowest box-count is defined as an optimization problem and can therefore be guided by an algorithm with a better convergence rate (Section 2.3.1). The box-count is treated as an objective function, defined over grid placement variables (Figures 2C–E). Optimization algorithms can be used to efficiently find local function minima (Ruszczynski, 2006). The computational expense of reducing quantization error may thus be greatly reduced by using an optimization algorithm as compared to the brute force method, not least because the problem turns out to be well-bounded and thus a global optimum can be sought within a fairly confined space.

#### 2.3.1. Pattern search

Beginning with any arbitrary grid of box size *s*, we define an objective function *J* that returns the box-count for a grid of the same box size, but with the grid origin translated by *dx*, *dy*, and *dz*, and the plant rotated around each axis by θ_*x*_, θ_*y*_, and θ_*z*_, respectively. In the following explanations, we will often denote the vector of choice variables

(2) $$\begin{array}{l} \begin{array}{c} v=\left(\begin{array}{c} dx\\ dy\\ dz\\ \theta_{x}\\ \theta_{y}\\ \theta_{z} \end{array}\right) \end{array} \end{array}$$

and its components *v*_*i*_, *i* = 1..6. Due to their effect on grid placement, we will refer to *v*_*i*_, *i* = 1..3 as the vector's translational components and *v*_*i*_, *i* = 4..6 as its rotational components.

The box-count exhibits discrete translational symmetry along each axis at intervals of *s* (i.e., for any integer *a*, translation of a grid of box size *s* along any axis by a distance *as* yields the original grid) and discrete rotational symmetry around each axis at intervals of $\frac{\pi }{2}$. We thus solved the constrained optimization problem

(3) $$\underset{dx,dy,dz,\theta_{x},\theta_{y},\theta_{z}}{\text{minimize}}N_{\epsilon }=J\left(\begin{array}{c} dx\\ dy\\ dz\\ \theta_{x}\\ \theta_{y}\\ \theta_{z} \end{array}\right)\text{ s}.\text{t}.\;\begin{array}{c} 0\leq dx\leq 1\\ 0\leq dy\leq 1\\ 0\leq dz\leq 1\\ 0\leq \theta_{x}\leq 1\\ 0\leq \theta_{y}\leq 1\\ 0\leq \theta_{z}\leq 1 \end{array}$$

having normalized all the translation components of the choice variable vector by *s* and the rotation components by $\frac{\pi }{2}$.

As the objective function is not continuously differentiable, classical gradient-estimation methods cannot be used. Instead, we employed pattern search (PS, Audet and Dennis, 2003, see Figures 2F–H for illustration) as implemented in MATLAB's native patternsearch() function. This iterative method begins by evaluating the objective function at a chosen *starting point*, i.e., a chosen set of values for the entries in the choice variable vector. At each iteration, PS evaluates the objective function at points that are a given interval Δ*v* away in both directions along each of the choice variable vector dimensions *v*_*i*_, *i* = 1..6. If this poll is successful, i.e., if *J* (*v*_*i*_ ± Δ*v*) < *J* (*v*_*i*_) for at least one *i*, then the next iteration begins from the point where the minimum value for *J* was found; otherwise, the interval Δ*v* is reduced. The search continues until a convergence criterion is met (e.g., until Δ*v* < τ, where τ is a chosen tolerance value, but the MATLAB function employs multiple convergence criteria). For best results using MATLAB's patternsearch(), the rotation components of the choice vector had to be rescaled to match the translation components, since a single Δ*v* is applied in all dimensions at each step.

In order to search for a global, rather than local optimum grid, the domain can be covered with *multiple* starting points, and the PS method is then run beginning at each one. Increasingly dense sets of starting points should result in the discovery of increasingly lower local minima. Any number of algorithms for choosing sets of starting points may be followed. We used an algorithm that preliminary trials showed yielded a reasonable tradeoff between minimizing the box-count and increasing computational cost. In this algorithm, separate rules governed the choice of translational and rotational components. The number of points along translational dimensions of the choice vector remained constant: all eight combinations of the values *v*_*i*_ = 0 $\text{and}\;v_{i}=\frac{1}{2}s,i=1..3$ (before normalization) for the translational components were always used. The overall density of starting points was increased by combining these with an increasing number of values for the rotational components at subsequent steps of the algorithm. We used *k* evenly spaced points on the rotational interval $\left[0,\frac{\pi }{2}\right)$ in each rotational dimension at the *k*th step, for a total of *k*^3^ rotations and 8*k*^3^ starting points. At the first step of the algorithm, all rotational components take the value (before normalization) $v_{i}=\frac{\pi }{4},i=4..6$ and there is a total of 8 points. At the second step, all combinations of values $v_{i}=\frac{\pi }{6}$ and *v_i_* = $\frac{\pi }{3}$, *i* = 4..6 are used for the rotational components and there is a total of 64 points, and so on.

With increasingly dense sets of starting points, search path redundancy is increasingly likely at higher steps of this algorithm, resulting in an unproductive increase in computational cost. In order to eliminate this effect, the algorithm was supplied with a stopping function, which terminated execution after any iteration that reached a point in the choice variable domain that had previously been encountered at the same Δ*v* value—the “history” function. PS was executed both with and without this function and the computational costs of the two versions were compared.

#### 2.3.2. Brute force

As a reference for the cost-efficiency of PS, the brute force (BF) method was also implemented. Box-counts were executed at individual points within the choice variable domain. The same vector of choice variables for transforming the grid [see Equation (2)] was used as in the PS method, but, instead of an optimization approach, the algorithm simply chose increasingly dense sets of points systematically in a series of steps.

Separate algorithms were used to subdivide the translational and rotational subdomains, based on preliminary results that showed slightly different means of subdivision to be most effective at achieving lower box-counts in each subdomain.

At step 0, only the point (0, 0, 0) of the translational subdomain (*v*_*i*_, *i* = 1..3) was used. At subsequent steps, each subinterval in every dimension of the subdomain was bisected by new points, so that at step 1 all combinations of *v*_*i*_ = 0 and *v_i_* = $\frac{1}{2}$*s*, *i* = 1..3 were used, at step 2 all combinations of $v_{i}=0,\frac{1}{4}s,\frac{1}{2}s,\text{and}$ $\frac{3}{4}$*s*, *i* = 1..3 were used, and so on. Thus, at the *k*^th^ step of translation, box-counts were conducted at 8^*k*^ grid positions that were distributed evenly through the subdomain. The rotational subdomain (*v_i_*, *i* = 4..6) was subdivided as in the algorithm for finding starting points for the PSmethod, described above.

To assess how grid position and orientation affect box-count results separately, while taking into account our computational power constraints, the sets of points obtained for the two subdomains were combined such that, for the first four steps of rotation (with 1, 8, 27, and 64 separate orientations, respectively), the first six steps of translation were implemented, and minimum box-counts were recorded for each combination.

### 2.4. Implementation and analysis

Estimated QE $\left(\hat{\epsilon }\right)$ was found for each box-count as the difference $\hat{\epsilon }=N_{\epsilon }-\min\left(N_{\epsilon }\right)$, where *N*_ϵ_ is the box-count found for a plant at a given grid size by a given iteration of a method and min(*N*_ϵ_) is the minmum count achieved for a plant at a given grid size, i.e., for all configurations of both the BF and the PS methods. Each of the methods was considered cumulative, such that both cost and minimum box-count at a given step reflected the results from all previous steps as well, while avoiding double-counting the costs of duplicate calculations in the BF method.

FD estimates were found in two separate ways. In order to match the most commonly used method, we found the box-count dimension by linear regression on the log(*N*) vs. log(1 ∕ *s*) data. We also used Reeve's (1992) method of differences, finding local slope estimates for the log(*N*(*s*)) ∝ log(*s*) relationship using the finite difference numerical approximation

(4) $$\begin{array}{l} V_{i}=\frac{\log\left(N_{i+1}\right)-\log\left(N_{i}\right)}{\log\left(s_{i+1}\right)-\log\left(s_{i}\right)} \end{array}$$

at grid sizes *s*_*i*_, *i* = 1, 2, 3, 4 and finding their mean, $\bar{V}$.

For each iteration of each method, we evaluated the effect of QE on FD calculations by noting the minimum achievable relative error in the FD estimate, as compared to the value found with the best available box-counts.

Statistical self-similarity was assessed on the coarse root systems using best-available box-counts. Following from the estimation by regression, one-sided median tests (Gibbons and Chakraborti, 2003) were used on regression residuals pooled for each scale to test the hypothesis that they come from a distribution with median 0. Also, a quadratic model was fit to the pooled residuals, as well as the residuals of each plant separately, and the significance of the second-order term was evaluated using a *t*-test.

For the local estimator, we tested the null hypothesis that the local slope estimates of each plant have a single common mean (the plant's true box-count dimension) and variance that can be estimated from the data, i.e., *H*_0_ : *V*_*i*, *j*_ ~ *N* (*D*_*j*_, σ_*j*_) for *i* = 1..4 local estimates and *j* = 1..36 plants, which follows in Reeve's (1992) method of differences from the assumption that log(*N*(*s*)) ∝ log(*s*). We used the Games-Howell test for one-way ANOVA (Games and Howell, 1976) on local slope data pooled for all 36 plants to do pairwise comparisons of the means of local slopes in four groups by scale. The Games-Howell test has lower power than classic ANOVA, but is designed specifically for groups with non-homogeneous variances and was used because the variance was observed to increase with scale.

The ANOVA test relies strongly on the central limit theorem. We thus decided to supplement it with a test that takes seriously the null hypothesis that observations are taken from distributions with plant-specific means and variances, and assumes that they cannot be treated as approximately identically distributed at each scale. Under this assumption, we calculated the residuals $V_{i,j}-{\bar{V}}_{j}$ for each plant, and divided them by the estimated variance to find normalized residuals, which follow the standard normal distribution. We then performed one-sample *z*-tests on the normalized residuals to evaluate whether they actually come from a distribution with a mean of 0. This test relies on the assumption that the variance of local slope observations is well characterized for each plant.

We devised a final test that makes no extra assumptions on the residuals at all and can be used to evaluate the prevalence of statistical self-similarity within a set of objects whose local box-count slopes are measured at the same scales. Given the null hypothesis, the residuals are symmetrically distributed about 0, making the sign of each residual a Bernoulli variable with *p* = 0.5. The number of residuals in a set of *n* will have the same sign, then, is a random variable *T* ~ *Bin* (*n, p*). The likelihoods of our observations of *t* same signs in sets of residuals for all 36 plants at a given scale can thus be found as *P* (*T* ~ *Bin* (36, 0.5) ≥ *t*). This analysis can be taken one step further, to evaluate the greatest number of signs of residuals that can be considered to come from the binomial distribution (at a given significance level α) given the observation *t*. This entails finding the largest number *m*, such that *P* (*T* ~ *Bin* (*m, p*) = *n* − *t*) ≥ α. For example, 36 negative signs in a set of 36 yields a value of 4 at a significance level of α = 0.05, since *P* (*T* ~ *Bin* (4, 0.5) = 0) = 0.0625 and *P* (*T* ~ *Bin* (5, 0.5) = 0) = 0.0312.

All programming was done in MATLAB, © Mathworks (2014), and the functions were run as parallel jobs on Yale University's Grace cluster of 80 IBM NeXtScale nx360 M4 servers, each with 20 E5-2650 cores and 128 GB RAM. Computational cost (CPU time) scaled linearly with the number of box-counts executed for both the PS and BF methods. Initial trials found negligibly different rates of CPU time increase with box-count executions between the two methods and box-counting was taken to dominate the cost of both. Because it is conserved across platforms, the number of executed counts is thus used throughout as a measure of computational cost.

## 3. Results

### 3.1. Quantization error levels

Although greater absolute levels of quantization error $\hat{\epsilon }$ are observed at finer grid scales, the relative error, which is the absolute error in proportion to the minimum box-count value $\frac{\hat{\epsilon }}{\min N_{\epsilon }}$, is greater at coarser scales. This pattern can be seen in Table 1, which summarizes the statistics of estimated quantization error at all grid sizes for a representative plant (shown in Figure 3). The effect of QE on box-counts is thus greater in log-space at coarser grids, which is clear from a comparison of the distribution of the log of the box-counts *N*_ϵ_ to the log of the minimum box-counts min(*N*_ϵ_) in the table. Since the log of coarser scale box-counts increases more than finer scale ones, the expected effect of QE on the slope of the relationship is to decrease it, implying a negative bias in the box-count dimension estimate. In the case of the example plant, the slope decrease is from 1.42 for the minimum box-counts to 1.31 for the mean observed counts, which represents 7.4% error.

**Table 1:** **Summary of quantization error statistics for *n* = 32, 768 brute force grid translations of the *Berberis thunbergii* plant shown in Figure 3**.

**Grid size [cm]**	**1**	**2**	**4**	**8**	**16**
$\hat{\epsilon }$ ^1^	120.35 ± 53.12	84.51 ± 180.3	47.28 ± 73.60	31.85 ± 19.33	19.90 ± 9.16
$\frac{\hat{\epsilon }}{\min N_{\epsilon }}$ (%)^1^	5.10 ± 1.0e-3	7.68 ± 0.01	10.04 ± 0.03	19.79 ± 0.07	43.26 ± 0.43
log(*N*_ϵ_)^1^	7.82 ± 8.6e-5	7.08 ± 1.3e-4	6.25 ± 2.7e-4	5.26 ± 5.2e-4	4.19 ± 2.1e-3
log(min(*N*_ϵ_))	7.76	7.00	6.15	5.08	3.83

1 Observed mean (± variance) for a single representative orientation.

**Figure 4:**
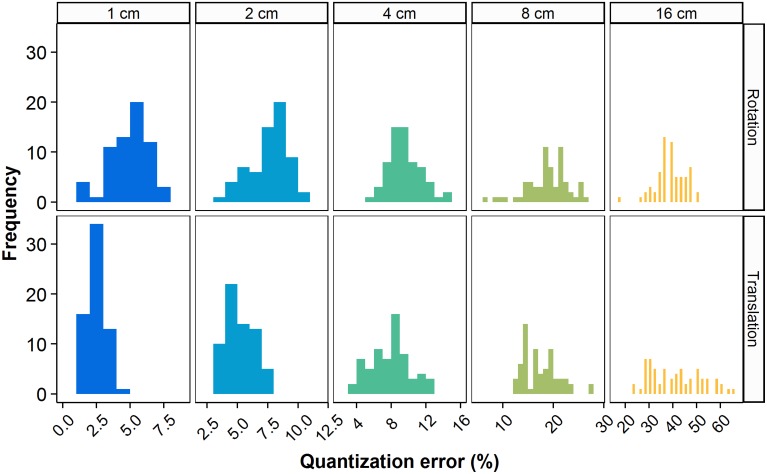
**Relative quantization error for the representative *Berberis thunbergii* plant shown in Figure 3**. Box-counts from the first 64 rotations (top) and the first 64 translations (bottom) are shown independently at each box size.

The separate effects of grid translation and rotation were of similar magnitude. Figure 4 shows the relative error under the first 64 translations and the first 64 rotations from the origin (independent of each other) of this same plant. While, locally, translation or rotation may have a greater or lesser effect at a given grid size for a given plant, we found no discernible general patterns that held across our dataset.

### 3.2. Method convergence

**Figure 5:**
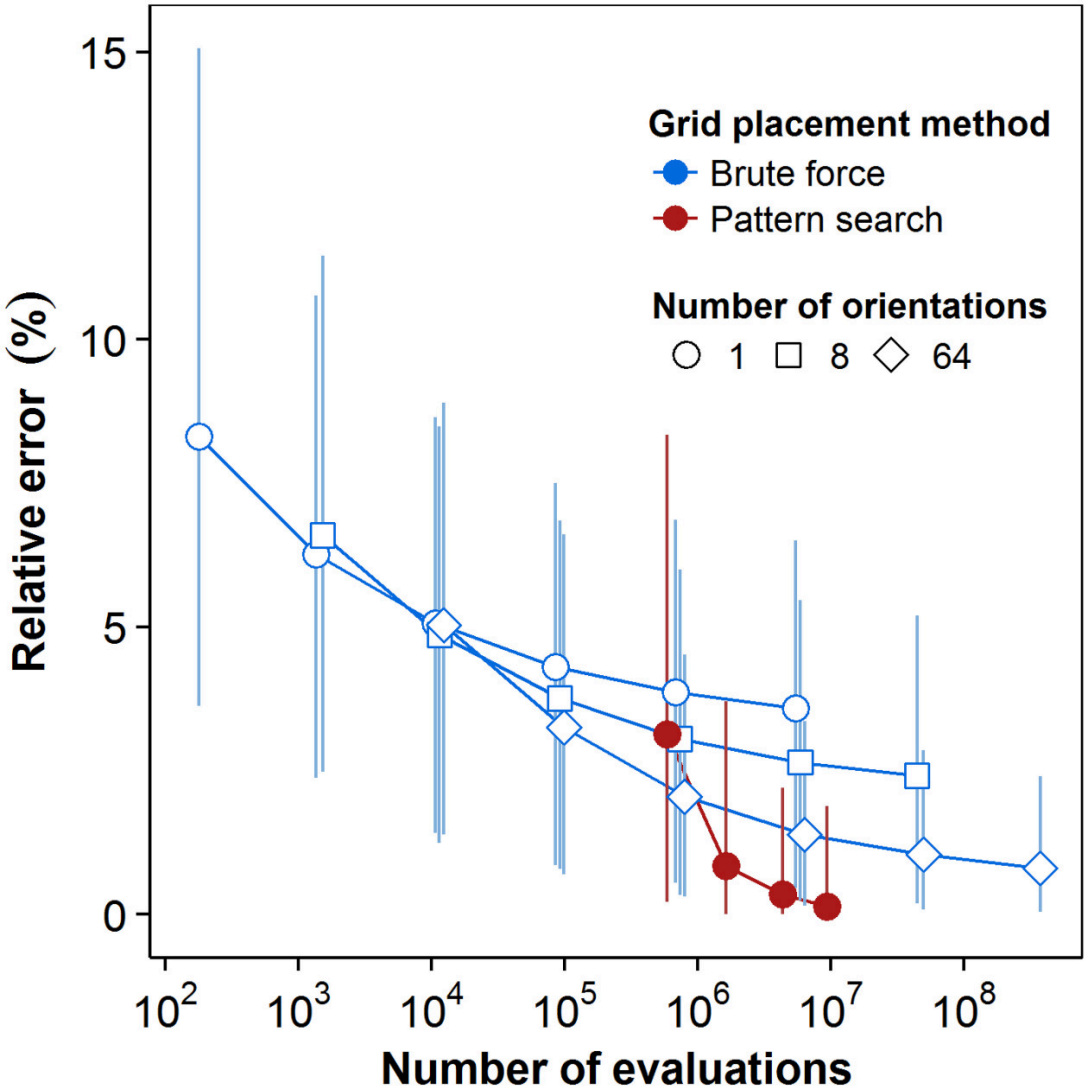
**Convergence plot for the brute force and pattern search methods**. Computational cost is quantified as the number of box-counts executed. Box-counts were pooled over all grid sizes and over all plants to reflect the total cost for the dataset. Error estimates reflect the lowest box-counts achievable for a given number of orientations and translations. Point markers represent the mean estimated relative error levels over all (*n* = 36) plants. Error bars indicate 95% confidence intervals, see text for details.

Quantization error strongly affected the results of FD estimation. Levels of the error decreased with increased computational power using either method, leading to lower errors in the FD estimates themselves. Figure 5 shows the rates of convergence of both BF and PS methods to the best-available FD estimates. For the BF method, the results for three series of steps of translation at a given step of rotation (as per the algorithm explained in Section 2.3.2) are shown; these are the series of 8^*k*^ translations for *k* = 0..5 successively, at 1, 8, and 64 rotations, respectively. Data were pooled for all 36 plants. In order to examine the expected value and confidence intervals, we exploited the fact that these results form nested datasets. For each nested configuration, we randomly selected up to 4096 box-counts from the full dataset for 64 rotations and 32,768 (or 8^5^) translations and took the mean and confidence intervals of the resulting sample set. This approach effectively enabled us to sample the translations of multiple grid origins and rotations from multiple initial grid orientations for each step of the BF method. No such sampling is possible for the PS method, so the distributions in these cases come exclusively from pooling data over all plants.

As Figure 5 shows, for a single grid with no attempt at rotation or translation (first point in the brute force series with a single orientation), the mean value of the relative error in the FD estimate due to quantization is 8.28% of the best available FD estimate. The error distribution has a long tail, however, and the relative error at the high end of the 95% confidence interval is 15.1%. In the BF method, translation alone reduced the mean normalized error by 56.8% over the range of computational cost explored. Series of the BF method with greater numbers of orientations started at greater initial cost and similar or higher mean error, but converged to lower mean levels of error over the same range. Comparing the series with no rotation to that with 64 orientations at the highest cost achieved by translation alone (5.9e6 box-count evaluations), mean error was 3.59% for the former and 1.39% for the latter. The 64-orientation series also retained faster convergence at higher costs: 32% reduction of mean error between algorithm steps compared to 7% for the single orientation. Greater numbers of orientations also reduced the extent of the 95% confidence interval at comparable costs. At that same cost level, translation alone reduced the upper limit of the interval to 6.5%, while the series with 64 orientations reduced it to 3.36%.

PS had a greater initial cost, simply because the PS algorithm executes the box-count more than once from each starting point. With the lowest number of starting points, both the mean error and the upper limit of the 95% confidence interval are higher for PS than for BF results at comparable cost. There is, however, also a greater convergence rate for both the average value and the top boundary of the 95% confidence interval. The mean relative error in FD estimation using PS dropped over 95% from 3.14% to 0.13% over a cost interval from 5.9e5 to 9.4e6 box-counts. The last increase of the number of starting points yielded a drop in the resulting mean relative error of 61%, indicating continuing convergence at higher intensities of search for the global optimum. The upper limit of the confidence interval dropped to 1.9% over the whole range of cost, while its lower end reached 0, reflecting the fact that the PS estimates were the best available estimates for an increasing number of plants.

### 3.3. History function time savings

**Figure 6:**
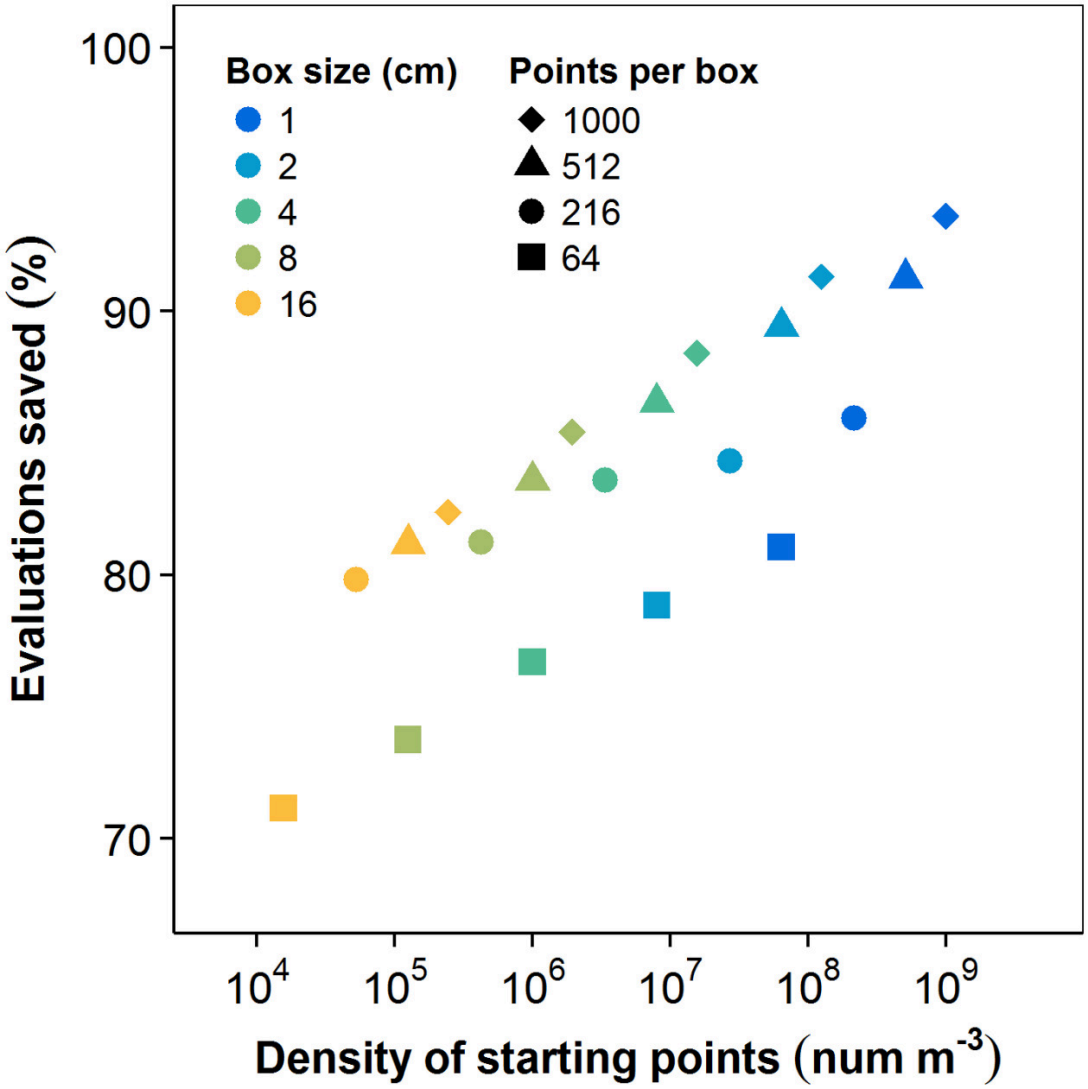
**Box-count evaluations eliminated by including the history function, as a function of the starting point density ($n_{pts}∕s^{3}$)**. The density of starting points, and thus the time savings due to elimination of redundancy, increase separately with increasing number of starting points and decreasing box size.

The history function significantly reduced the computational cost of PS. Numbers of box-count evaluations were aggregated for all (*n* = 36) plants at each number of PS starting points and for each box size. The difference between the number of box-count evaluations in PS with and without the history function are shown in Figure 6 as a percentage of the number of evaluations in the naïve method. Both the number of start points and grid resolution affect the spatial density of starting points and, thus, the likelihood of path redundancy. Accordingly, savings increased with both factors. For the largest box size and fewest starting points, the history function saved 71.1% of the naïve method's box-count evaluations. The highest observed savings attained 93.6%. The convergence rate of PS (per unit cost) is thus strongly dependent on the use of this history function.

### 3.4. FD estimates

The box-count dimension values we found with the best-available box-counts are summarized in Table 2. There was little difference between the values of the mean slope estimator due to Reeve (1992) and the more widely used regression estimator. The main advantage of the mean slope estimator, of course, lies not in its ability to obtain more “accurate” values, but in its ability to correctly characterize the error in each individual estimate. We found no significant differences in box-counting dimension among species using one-way ANOVA [*F*_(5, 30)_ = 2.1, *p* = 0.093].

**Table 2:** **Summary of box-count dimension statistics**.

**Species**	**Mean slope est.^1^**	**95% C.I. bounds**	**Regression est.^1^**	**95% C.I. bounds**
*Viburnum dentatum*	1.318 ± 4.13%	1.24–1.40	1.311 ± 4.07%	1.23–1.39
*Rubus allegheniensis*	1.211 ± 8.69%	1.10–1.39	1.207 ± 8.47%	1.10–1.38
*Lindera benzoin*	1.230 ± 6.43%	1.09–1.31	1.223 ± 6.32%	1.09–1.31
*Berberis thunbergii*	1.329 ± 4.78%	1.23–1.42	1.326 ± 4.80%	1.23–1.41
*Lonicera maackii*	1.253 ± 4.71%	1.16–1.32	1.248 ± 4.36%	1.16–1.31
*Rubus phoenicolasius*	1.289 ± 8.14%	1.15–1.41	1.262 ± 7.86%	1.15–1.40
All	1.269 ± 6.79%	1.09–1.42	1.264 ± 6.63%	1.09–1.41

1 Mean ± coefficient of variation.

### 3.5. Statistical self-similarity assessment

**Figure 7:**
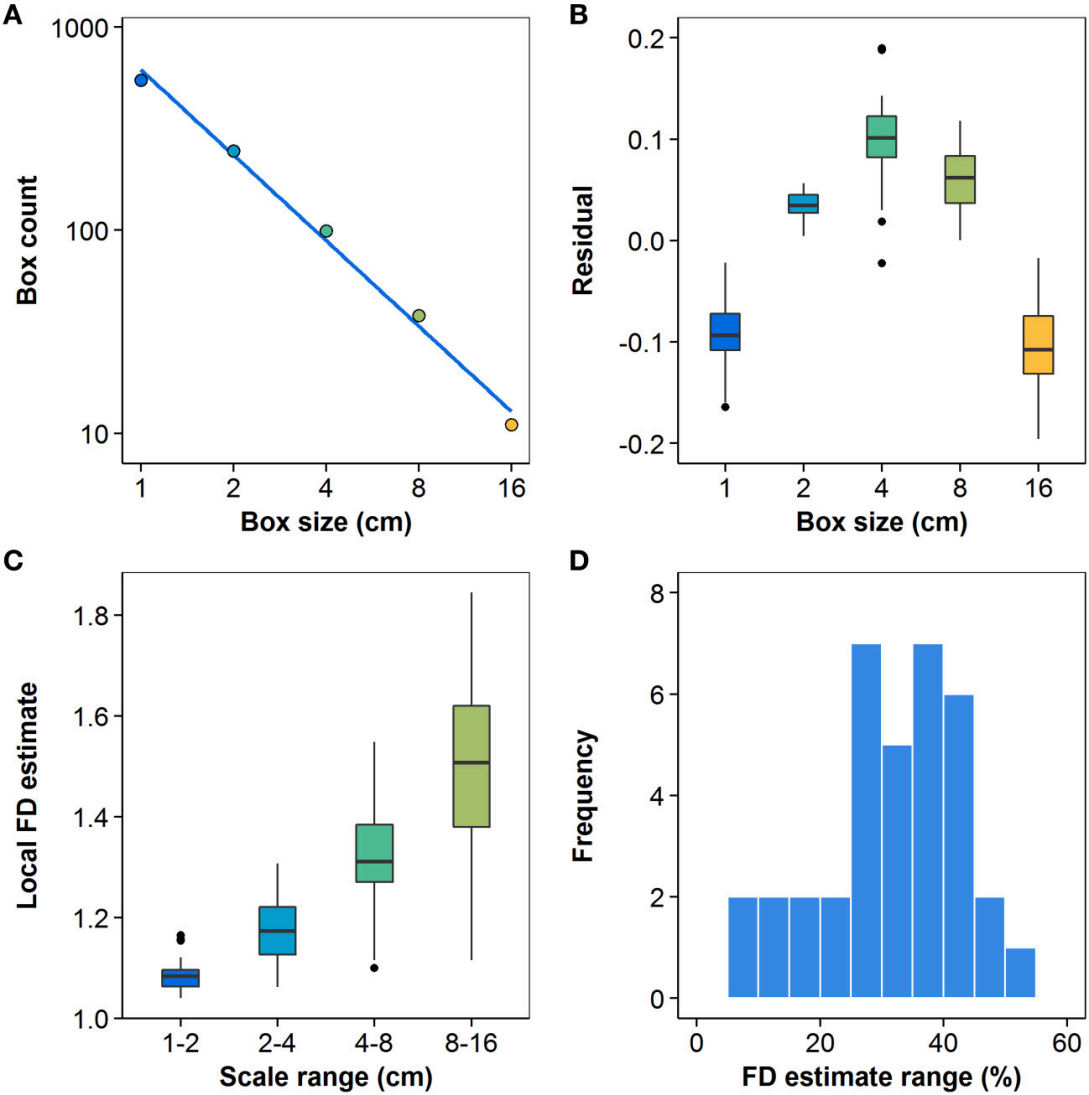
**(A)** Log-space linear regression (*R*^2^ > 0.99) to box-count data for the representative *Berberis thunbergii* plant shown in Figure 3; **(B)** distributions of residuals for all *n* = 36 plants at each box size; **(C)** local values of slope *V*_*i*_ from Equation (4); **(D)** distribution of differences between maximum and minimum local slope estimates, relative to a plant's mean FD estimate (*n* = 36). All box plots show the median, 25th and 75th percentiles, whiskers extending to 1.5 times the inter-quartile range, and outliers.

The degree of statistical self-similarity was first assessed by rigorously examining the quality of the regressions that yielded the most commonly-used estimator. Figure 7A shows the log-space linear regression to the best available box-count data for the *Berberis thunbergii* plant shown in Figure 3, from which its FD was estimated. While the line fits the data very well (*R*^2^ > 0.99), which might lead one to prematurely conclude that statistical self-similarity is present, a bowed pattern can be observed in the residuals between the data and the linear model. Figure 7B shows the distributions over all plants (*n* = 36) of such residuals and indicates clearly that in no case are they distributed normally with a mean of 0. Such a hypothesis was rejected by one-sided median tests for each grid size individually (*p* < 0.001). Instead, they exhibit a clearly non-linear pattern, both visually and statistically. The second-order coefficient of a quadratic polynomial fit to the residuals is significantly different from 0 (*p* < 0.001). All but three of the plants also exhibit this quadratic pattern on an individual basis (*p* < 0.04). The remaining three all appear as outliers in Figure 7B at the 4 cm box size. Two are high outliers and their residuals exhibit a V-shape, as opposed to a parabolic shape. This is just as problematic for the regression. The last is a low outlier and exhibits a W pattern over all five grids. It alone allows for the possibility of statistical self-similarity.

The distributions of local slopes over four different ranges of scales for the 36 plants are shown in Figure 7C. The clear visual trend toward higher slopes at coarser grid resolutions is in keeping with the pattern of regression residuals. The trend is also confirmed by one-way ANOVA with Games-Howell *q*-tests, which found significantly different means (*p* < 0.01) for all pairwise comparisons of the four scale ranges. The *z*-test for a standard normal distribution using normalized residuals also rejected the hypothesis of a zero mean for all scale ranges (*p* < 0.01) except the third (4–8 cm), effectively showing the local slope values for each plant do not have a common mean. The residuals of the mean local slope estimates at the four scales had 36, 35, 4, and 0 negative signs, in order from finest to coarsest scale. These or more extreme observations have a likelihood of *p* < 0.001 under the null hypothesis, which can thus be rejected. The greatest number of residuals at each scale that may conform to the null hypothesis at a 5% significance level are 4, 8, 15, and 4, respectively. All tests thus support the view that statistical self-similarity is not prevalent in the present dataset. Despite the high *R*^2^ values found, the assumption of statistical self-similarity is not supported for the vast majority of the MTG digitizations in this study, even within the limited range of scales over which box-counting may appropriately be applied to them.

Figure 7D shows the distribution of the differences between the maximum and minimum local slopes, relative to their mean FD estimate, over all root system representations. That is, the histogram shows the number of plants whose rate of scaling changes by a given proportion of its mean FD over the measured range of scales. For only four of the plants is this difference less than 7.9% of the mean FD estimate. The difference exceeds 25% of mean FD for three quarters of the studied plants. Not only is the majority of MTG digitizations examined here not self-similar, they do not even admit of a first approximation FD estimate that would be valid over the relevant range.

## 4. Discussion

### 4.1. Quantization error

Quantization error had a clear and significant effect on FD estimates. In this study, both box-counts and log-space slope estimates showed significant estimated error when quantization was not taken into account. Because QE is strictly positive, and because it tended to produce more significant relative error (and thus log-space error) in the box-counts at coarser scales, it most commonly led to a negative bias in slope estimates regardless of the estimator. This stands in contrast to the effect of QE in pixelated images, found by Gonzato et al. (2000) to be predominantly positive bias. The difference was likely due to the smooth nature of the underlying object in our study, namely the linear interpolant. Regardless of its direction, the effect is significant in both cases. In our study, it took box-counts at 512 or more placements (for each grid size, for each plant) to reduce the top of the 95% confidence interval to below the coefficient of variation (CV) of FD over all the plants.

Da Silva et al. (2006) proposed mitigating the influence of quantization error by narrowing the range of box sizes used in FD estimation, eliminating the finest grids. This approach has little or no computational cost and should be used whenever possible. Unfortunately, when attempted with our dataset (even on the coarsest grids), the approach of Da Silva et al. (2006) did not yield a sufficient decrease in quantization error with our data (error in FD estimates below their CV was deemed acceptable). Moreover, the approach would have significantly narrowed the range of grid sizes available for the estimation. The range of grid sizes was already quite small, as the coarse root system digitizations did not span more than two orders of magnitude, and any further reduction in the range of measurement scales would be highly undesirable (Halley et al., 2004).

Like Gonzato et al. (2000), we found that grid orientation plays a significant role in determining the magnitude of QE. This is significant because most of the studies in our review that attempted to reduce QE only explored grid translations. Our finding is contrary to the observations of Da Silva et al. (2006), who found, using BF, that grid orientation did not lead to significant QE. In our work, however, BF exhibited apparent convergence at higher levels of error when using fewer orientiations. At lower investment of computational resources, adding more grid orientations did little to improve FD estimates. At higher levels of computational cost, additional orientations clearly improved the sampling of the QE space, given that they led to significantly lower confidence intervals and higher rates of convergence. Both apparent convergence and insufficient computational resources may have played a role in the results of Da Silva et al. (2006). On the other hand, that study compared box-count dimension values to FD estimates obtained by Boudon et al. (2006) for the same plants using the two-surface method (Zeide and Pfeifer, 1991), which (assuming the comparison is valid) supports the validity of its conclusions. Furthermore, the two studies investigated fundamentally different objects in different representations, and our results may thus not be comparable.

### 4.2. Pattern search vs. brute force

BF provides box-counts with low confidence at low cost, but its inefficient way of sampling the domain makes it an infeasible means of achieving satisfactory high-confidence FD estimates. The convergence rate of PS is much greater than that of the BF method. It has higher upfront costs, but converges on lower box-count values with lower total costs. It was the only way to get the 95% confidence interval of relative FD error below one third of the CV for FD and the most efficient means of reducing the mean error level to an order of magnitude less than the CV. It offered the greatest chance of efficiently eliminating quantization error altogether, which is a prerequisite of unbiased FD estimation. A crucial component of the PS method that allows for this higher rate of convergence is avoiding search path redundancy by incorporating a history function in the global optimum search. We found that different ways of scaling the choice variable vector also yielded different levels of computational efficiency. We did not investigate the effects of different polling or searching methods within PS on the convergence rate; these may be found to improve algorithm efficiency further still.

Pattern search is not applicable only to root systems, but to any object whose FD is to be estimated by box-counting. It is especially relevant for objects that do not span a great range of scales and for which the scale cut-off solution of Da Silva et al. (2006) may thus be impractical. This may be true of many root systems and other plant structural or anatomical features. As increasingly powerful computers become increasingly widespread, the initial cost hurdle of PS will become less significant and this tool will become more easily accessible to researchers.

### 4.3. Statistical self-similarity of the coarse root system digitizations

The fact that regression residuals were not distributed symmetrically about 0, but had a bowed pattern that was fit by second-order polynomials with statistically significant quadratic terms, underlines the danger of relying on *R*^2^ alone to evaluate the fractal model. Slope estimates differed significantly by scale and we were able to show that the local slope estimates for the great majority of plants do not have a common mean across scales. This is an explicit assumption of the Reeve (1992) method of differences, whose mean slope estimator is an approximation of the common mean of local slope estimates. The fact that the local slope values in our dataset do not share a common mean across scales allows us to reject the idea that the box-count data follow a linear model strictly—there is no true mean rate of emergence of detail at increasingly fine scales.

Under the pragmatic view of statistical self-similarity, we might still estimate a box-count dimension in the absence of a true mean, so long as the estimated apparent mean provides a reasonable approximation for the scaling properties of the digitizations over some range. In our dataset, however, the range of local slope estimates spanned over 25% of the FD estimator for three quarters of the plants, and the variation of the local slope estimates across scales far exceeded variation in box-count dimension estimates between plants within a species, which was, in turn, about the same as between species. The FD estimates thus seem neither representative nor informative.

One solution to a lack of general representativeness of the FD estimate in the literature has been to narrow the range of regression, yielding a box-count dimension more representative locally, i.e., at a particular scale (Lontoc-Roy et al., 2006; Dutilleul et al., 2015). This may, however, run afoul of the convention for a minimum scale range when trying to quantify a scaling coefficient (Hamburger et al., 1996; Halley et al., 2004). In other words, narrowing the range may be a legitimate pragmatic solution, but only if we take care not to essentially fit a tangent to the curve with little representativeness, except at a point. In the case of our data, using a single value of box-count FD would misrepresent how detail emerges upon magnification of the coarse root systems; instead of a true or apparent mean rate of scaling of detail, a significant and lawful variation was clearly observed, with greater slopes at coarser scales.

Rather than the box-count dimension of a fractal model, what our regression residuals and local slope estimates reveal is a consistent tendency of the slope to approach 1 as *s* → 0. In other words, over the entire range of investigation, the ratio log(*N*) ∕ log(1 ∕ *s*) converges on the topological dimension of the finite sets. This cannot be rectified by moving the range upward, since we soon encounter the well-known upper limit of the range (Foroutan-pour et al., 1999; Halley et al., 2004), where the box size approaches one quarter of the object size for some of the plants. The range of scales at which our digitizations exhibit detail thus has no sub-range where a slope of the log(*N*) vs. log(1 ∕ *s*) relationship could be found that is not already converging on 1. Given that convergence of the slope to 1 is already present at the coarsest usable scales, it is clear that the digitizations exhibit detail at too few scales to be well approximated by a fractal model. This allows us to conclusively reject a linear approximation to our log(*N*) vs. log(1 ∕ *s*) data over the entire applicable range of *s*, meaning that our root system digitizations do not conform to our definition of statistical self-similarity (Section 1.2). This includes a rejection of apparent fractality for our digitizations and thus shows the limits of pragmatism when evaluating the statistical self-similarity of an object.

It must be kept in mind that the MTGs in our dataset include only the coarse portions of the root systems (diameter >2 mm). This data limitation is inherent in our measurement method, as the loss of fine roots and some finer-scale details is expected during digitization, and is common for such datasets. Our analysis thus cannot be used to support conclusions about the root system as a whole. Detail at scales of the coarse root systems is, however, well represented and we can conclude that the coarse root systems of the plants in our dataset do not exhibit statistical self-similarity, due to a lack of detail over a sufficient range of scales. The implication for the importance of stochastic reiterative growth in their development is that while the systems may be partly the result of reiteration, they are not sufficiently ramified for their overall form to be well approximated by a fractal model. We cannot exclude the possibility that if fine roots were part of the digitized representations, statistical self-similarity would be better supported (at least due to apparent fractality); however, it is not a foregone conclusion either.

A means of escaping the limitation inherent our data would be using simulated root systems. While such an approach might not reveal much about the statistical self-similarity of any particular real root systems, it would provide a means of testing the hypothesized link between repetitive branching and statistical self-similarity. Depending on the outcome of such a study, a model that includes a combination of a repetitive branching algorithm and the influence of environmental conditions might be used to evaluate the influence of each in the generation of root systems that exhibit a given degree and mode of deviation from statistical self-similarity. Given the imperfect correspondence between some of the related fractal concepts, however, such a study would have to proceed with caution.

Our statistical self-similarity analysis mainly serves as a methodological caution against freely assuming statistical self-similarity. As we have shown, statistical self-similarity can be a rigorously defined concept, formulated as a testable hypothesis to be rejected, but cannot be confirmed by regressions with a high *R*^2^. A lack of detail over a sufficient range of scales is a particularly difficult obstacle to statistical self-similarity, because it precludes the emergence of even apparent fractality in an object. This should raise doubts in the minds of researchers who aim to estimate FD for any relatively undeveloped root structure, whether it is only the coarse root portion (e.g., Oppelt et al., 2000), relatively young roots (Lontoc-Roy et al., 2006), or any root system representation whose box-count data show the same bowed pattern as ours (Eshel, 1998; Dzierzon et al., 2003). Put differently, a high *R*^2^ in box-count regressions does not guarantee even apparent fractality. The representativeness of a FD estimate should be evaluated over a sufficient range of scales and if the estimate is not found to be informative, Euclidean alternatives (such as root length density or branching frequency) should be considered for use in any subsequent analysis, as they may better capture the complex geometry of the object.

Finally, we note that some studies, finding no (statistical) self-similarity in the root systems examined, concluded that they exhibit *bi-* (Dutilleul et al., 2015) or *multi-fractal* (Berntson and Stoll, 1997; Ketipearachchi and Tatsumi, 2000) properties instead. A multifractal is a measure of an object that exhibits power-law scaling, but whose different portions follow different scaling parameters. The proposition that a root system has multifractal measures cannot be tested with the simple box-counting procedure used here and lies outside of the scope of the present study. Even if root systems are not statistically self-similar, however, they are not automatically multifractal. That is a second positive empirical assertion, which ought to be tested separately. The coarse root systems in our dataset are not best described as multifractals, but simply as objects lacking detail over a sufficient range of scales, whose scaling parameters therefore vary as a function of scale.

## Author contributions

MB conceived the study, performed the bulk of the analysis and led manuscript composition. JC built the root system data set and contributed to manuscript composition and revision. JS contributed to study design, data analysis and interpretation, as well as manuscript revision.

## Funding

The computational portion of this work was supported in part by the facilities and staff of the Yale University Faculty of Arts and Sciences High Performance Computing Center and the data-collection by USDA-NIFA grant no. 2009-35900-06016.

### Conflict of interest statement

The authors declare that the research was conducted in the absence of any commercial or financial relationships that could be construed as a potential conflict of interest.
