# Supplementary material for: Box-Counting Dimension Revisited: Presenting an Efficient Method of Minimizing Quantization Error and an Assessment of the Self-Similarity of Structural Root Systems
Source: Front Plant Sci. 2016 Feb 18;7:149. doi: 10.3389/fpls.2016.00149 (PMC4758026; doi:10.3389/fpls.2016.00149)
Supplement: Supplementary file 1 [file Table1.pdf]

frontiers

Supplementary Material: Box-counting dimension revisited: presenting an efficient method of minimising quantisation error and an assessment of the self-similarity of structural root systems

Martin Bouda*, Joshua S. Caplan and James E. Saiers

*Correspondence:
Martin Bouda
martin.bouda@yale.edu

SUPPLEMENTARY TABLE 1

Summary of plant box-counting studies, with a focus on root systems, indicating how self-similarity was supported in each study and how quantisation error was dealt with.

Reference	Object	Suggested utility of FD	Accounts for quantisation error	Support for self-similarity
Tatsumi et al. (1989)	Root systems of crop species	Characterise branching and morphology in a simple and quantitative way	No	Quality of fit by linear regression (R^2, p-value)
Eghball et al. (1993)	Root systems of *Zae mays* L.	Quantify branching intensity in relation to resource (N) acquisition	No	By assumption
Lynch and Vanbeem (1993)	Root systems of different *Phaseolus vulgaris* L. genotypes	Characterise branching intensity; theoretical implications for adaptation	No	By assumption
Berntson (1994)	Tracings of *Betula spp.* roots	Methodological discussion	No	By assumption
Berntson and Stoll (1997)	Mathematical fractals and *Solidago altissima* L. root systems	Quantify space-filling properties of structures	No	Tested via curvilinearity of regression residuals; nonlinearity found
Nielsen et al. (1997)	Simulated roots	Quantify root system architecture in regard to mechanical and resource acquistion functions	No	By assumption

Summary of plant box-counting studies, with a focus on root systems, indicating how self-similarity was supported in each study and how quantisation error was dealt with.

Reference	Object	Suggested utility of FD	Accounts for quantisation error	Support for self-similarity
Eshel (1998)	Root systems of *Lycopersicum esculentum* Mill.	Quantify complexity and branching characteristic	No	By assumption
Masi and Maranville (1998)	Root systems of *Sorghum bicolor* L. genotypes	Differentiate root systems of different genotypes	No	By assumption
Nielsen et al. (1999)	Root systems of *Phaseolus vulgaris* L.	Evaluate space-filling and thus soil exploration as a means of addressing P acquisition	No	By assumption
Oppelt et al. (2000)	Coarse root systems of African tree species	Elucidate architectural and life-history strategies	No	Quality of fit by linear regression (R^2)
Ketipearachchi and Tatsumi (2000)	Root systems of six legume species	Characterise root system morphology and architecture	No	Disregarded
Izumi and Iijima (2002)	Root systems of *Manihot esculenta* Crantz	Understand root habit for application in intercropping systems	No	By assumption
Dzierzon et al. (2003)	*Pinus sylvestris* L. stems, real and simulated	Quantify space-filling properties	No	By assumption
Bari et al. (2004)	Root systems of *Olea europaea* L. cultivars	Link FD to water use efficiency	No	By assumption
Walk et al. (2004)	Simulated root systems	Evaluate efficiency of soil exploration	No	By assumption
Dannowski and Block (2005)	Root systems of entire plant communities	Quantify complexity with a view to structural efficiency, water and nutrient flows	No	By assumption, due to repetitive branching
Lontoc-Roy et al. (2005)	Root systems of *Zea mays* L.	Quantify complexity with a view to characterising development	Brute force: 12 transl.	Quality of regression (R^2)
Da Silva et al. (2006)	Digitised and simulated canopies of *Prunus persica* L. foliage, cantor dust	Methodological study	Scale cut-off	By assumption
Lontoc-Roy et al. (2006)	Root systems of *Zea mays* L.	Quantify complexity	Brute force: 12 transl.	Multiple scale ranges to maximise R^2

Summary of plant box-counting studies, with a focus on root systems, indicating how self-similarity was supported in each study and how quantisation error was dealt with.

Reference	Object	Suggested utility of FD	Accounts for quantisation error	Support for self-similarity
Dutilleul et al. (2008)	Skeletonised canopies of *Thuja occidentalis* L.	Link canopy complexity to light interception	Unclear	By assumption
Han et al. (2008)	Root systems of *Solanum tuberosum* L.	Measure effect of soil pathogen on space occupancy	Brute force	Quality of fit by regression (R^2)
Manschadi et al. (2008)	Root systems of *Triticum aestivum* L. genotypes	Quantify branching intensity with a view to resource acquisition and breeding for drought resistance	No	By assumption
Barto and Cipollini (2009)	Root systems of *Impatiens pallida* Nutt.	Quantify effect of invasive-removal techniques on subsequent colonisation	No	By assumption
Dibble and Thomaz (2009)	Shoots of aquatic plants	Quantify complexity of macroinvertebrate habitats	No	By assumption
Wang et al. (2009)	Root systems of *Oryza sativa* L. genotypes	Quantify architecture and drought response with a view to drought adaptation of crops	No	By assumption
Grift et al. (2011)	Root systems of *Zea mays* L.	Crop phenotyping	No	By assumption
Ferreiro et al. (2013)	Shoots of aquatic plants	Quantify complexity of macroinvertebrate habitat	Brute force, extent unclear	By assumption
Pierce et al. (2013)	Roots of *Salix nigra* Marsh.	Quantify effect of soil saturation (redox potential)	Brute force by random walk	By assumption
Aagaard and Hartvig (2014)	Plant community canopies	Assess complexity of canopies across vegetation types and successional stages	No	By assumption
Yang et al. (2014)	Shoots of *Robinia pseudoacacia* L.	Evaluate effect of mycorrhizal infection	No	By assumption and quality of regression (R^2)
Dutilleul et al. (2015)	Crowns of miniature coniferous trees	Link canopy complexity to light interception	Brute force, 8 transl.	Two scale ranges imposed to maximise R^2 fit
Subramanian et al. (2015)	Root systems of *Zea mays* L.	Assess the effects of salinity stress	Brute force	Quality of fit by regression (R^2)

REFERENCES

Aagaard, K. and Hartvigsen, G. (2014). Assessing spatial patterns of plant communities at varying stages of succession. *Applied Mathematics* 5, 1842–1851. doi:10.4236/am.2014.512177

Bari, A., Ayad, G., Martin, A., Gonzalez-Andujar, J., Nachit, M., and Elouafi, I. (2004). Fractals and plant water use efficiency. In *Thiking in Patterns: fractals and related phenomena in nature*, ed. M. Novak. 315–316. doi:10.1142/9789812702746_0029. 8th International Conference on Thinking in Patterns -Fractals and Related Phenomena in Nature, Vancouver, CANADA, APR 04-07, 2004

Barto, E. K. and Cipollini, D. (2009). Garlic mustard (alliaria petiolata) removal method affects native establishment. *Invasive Plant Science and Management* 2, 230–236. doi:10.1614/IPSM-09-011.1

Berntson, G. (1994). Root systems and fractals - how reliable are calculations of fractal dimensions. *Annals of Botany* 73, 281–284. doi:10.1006/anbo.1994.1033

Berntson, G. and Stoll, P. (1997). Correcting for finite spatial scales of self-similarity when calculating the fractal dimensions of real-world structures. *Proceedings of the Royal Society B-Biological Sciences* 264, 1531–1537

Da Silva, D., Boudon, F., Godin, C., Puech, O., Smith, C., and Sinoquet, H. (2006). A critical appraisal of the box counting method to assess the fractal dimension of tree crowns. In *Advances in Visual Computing, PT 1*, eds. G. Bebis, R. Boyle, B. Parvin, D. Koracin, P. Remagnino, A. Nefian, G. Meenakshisundaram, V. Pascucci, J. Zara, J. Molineros, H. Theisel, and T. Malzbender. vol. 4291 of *Lecture Notes in Computer Science*, 751–760. 2nd International Symposium on Visual Computing, Lake Tahoe, NV, NOV 06-08, 2006

Dannowski, M. and Block, A. (2005). Fractal geometry and root system structures of heterogeneous plant communities. *Plant and Soil* 272, 61–76. doi:10.1007/s11104-004-3981-2

Dibble, E. D. and Thomaz, S. M. (2009). Use of fractal dimension to assess habitat complexity and its influence on dominant invertebrates inhabiting tropical and temperate macrophytes. *Journal of Freshwater Ecology* 24, 93–102. doi:10.1080/02705060.2009.9664269

Dutilleul, P., Han, L., and Smith, D. L. (2008). Plant light interception can be explained via computed tomography scanning: Demonstration with pyramidal cedar (thuja occidentalis, fastigiata). *Annals of Botany* 101, 19–23. doi:10.1093/aob/mcm273

Dutilleul, P., Han, L., Valladares, F., and Messier, C. (2015). Crown traits of coniferous trees and their relation to shade tolerance can differ with leaf type: a biophysical demonstration using computed tomography scanning data. *Frontiers in Plant Science* 6. doi:10.3389/fpls.2015.00172

Dzierzon, H., Sievanen, R., Kurth, W., Perttunen, J., and Sloboda, B. (2003). Enhanced possibilities for analyzing tree structure as provided by an interface between different modelling systems. *Silva Fennica* 37, 31–44

Eghball, B., Settmi, J., Maranville, J., and Parkhurst, A. (1993). Fractal analysis for morphological description of corn roots under nitrogen stress. *Agronomy Journal* 85, 287–289

Eshel, A. (1998). On the fractal dimensions of a root system. *Plant, Cell and Environment* 21, 247–251. doi:10.1046/j.1365-3040.1998.00252.x

Ferreiro, N., Giorgi, A., and Feijoo, C. (2013). Effects of macrophyte architecture and leaf shape complexity on structural parameters of the epiphytic algal community in a pampean stream. *Aquatic Ecology* 47, 389–401. doi:10.1007/s10452-013-9452-1

Grift, T. E., Novais, J., and Bohn, M. (2011). High-throughput phenotyping technology for maize roots. *Biosystems Engineering* 110, 40–48. doi:10.1016/j.biosystemseng.2011.06.004

Han, L., Dutilleul, P., Prasher, S. O., Beaulieu, C., and Smith, D. L. (2008). Assessment of common scab-inducing pathogen effects on potato underground organs via computed tomography scanning. *Phytopathology* 98, 1118–1125. doi:10.1094/PHYTO-98-10-1118

Izumi, Y. and Iijima, M. (2002). Fractal and multifractal analysis of cassava root system grown by the root-box method. *Plant Production Science* 5, 146–151

Ketipearachchi, K. W. and Tatsumi, J. (2000). Local fractal dimensions and multifractal analysis of the root system of legumes. *Plant Production Science* 3, 289–295

Lontoc-Roy, M., Dutilleul, P., Prasher, S., Han, L., and Smith, D. (2005). Computed tomography scanning for three-dimensional imaging and complexity analysis of developing root systems. *Canadian Journal of Botany-Revue Canadienne de Botanique* 83, 1434–1442. doi:10.1139/b05-118

Lontoc-Roy, M., Dutilleul, P., Prasher, S. O., Han, L., Brouillet, T., and Smith, D. L. (2006). Advances in the acquisition and analysis of ct scan data to isolate a crop root system from the soil medium and quantify root system complexity in 3-d space. *Geoderma* 137, 231–241. doi:10.1016/j.geoderma.2006.08.025

Lynch, J. and Vanbeem, J. (1993). Growth and architecture of seedling roots of common bean genotypes. *Crop Science* 33, 1253–1257

Manschadi, A. M., Hammer, G. L., Christopher, J. T., and deVoil, P. (2008). Genotypic variation in seedling root architectural traits and implications for drought adaptation in wheat (triticum aestivum l.). *Plant and Soil* 303, 115–129. doi:10.1007/s11104-007-9492-1

Masi, C. and Maranville, J. (1998). Evaluation of sorghum root branching using fractals. *Journal of Agricultural Science* 131, 259–265. doi:10.1017/S0021859698005826

Nielsen, K., Lynch, J., and Weiss, H. (1997). Fractal geometry of bean root systems: Correlations between spatial and fractal dimension. *American Journal of Botany* 84, 26–33. doi:10.2307/2445879

Nielsen, K., Miller, C., Beck, D., and Lynch, J. (1999). Fractal geometry of root systems: Field observations of contrasting genotypes of common bean (phaseolus vulgaris l.) grown under different phosphorus regimes. *Plant and Soil* 206, 181–190

Oppelt, A., Kurth, W., Dzierzon, H., Jentschke, G., and Godbold, D. (2000). Structure and fractal dimensions of root systems of four co-occurring fruit tree species from botswana. *Annals of Forest Science* 57, 463–475. 2nd International Workshop on Functional-Structural Tree Models, Clermont Ferra, France, Oct 12-14, 1998

Pierce, S. C., Koontz, M. B., Pezeshki, S. R., and Kroeger, R. (2013). Response of salix nigra [Marsh.] cuttings to horizontal asymmetry in soil saturation. *Environmental and Experimental Botany* 87, 137–147. doi:10.1016/j.envexpbot.2012.10.003

Subramanian, S., Han, L., Dutilleul, P., and Smith, D. L. (2015). Computed tomography scanning can monitor the effects of soil medium on root system development: an example of salt stress in corn. *Frontiers in Plant Science* 6. doi:10.3389/fpls.2015.00256

Tatsumi, J., Yamauchi, A., and Kono, Y. (1989). Fractal analysis of plant-root systems. *Annals of Botany* 64, 499–503

Walk, T., Van Erp, E., and Lynch, J. (2004). Modelling applicability of fractal analysis to efficiency of soil exploration by roots. *Annals of Botany* 94, 119–128. doi:10.1093/aob/mch116

Wang, H., Siopongco, J., Wade, L. J., and Yamauchi, A. (2009). Fractal analysis on root systems of rice plants in response to drought stress. *Environmental and Experimental Botany* 65, 338–344. doi:10.1016/j.envexpbot.2008.10.002

Yang, Y., Tang, M., Sulpice, R., Chen, H., Tian, S., and Ban, Y. (2014). Arbuscular mycorrhizal fungi alter fractal dimension characteristics of Robinia pseudoacacia L. seedlings through regulating plant

growth, leaf water status, photosynthesis, and nutrient concentration under drought stress. *Journal of Plant Growth Regulation* 33, 612–625. doi:10.1007/s00344-013-9410-0
